# Human Milk Secretory Antibodies against Attaching and Effacing *Escherichia coli* Antigens

**DOI:** 10.3201/eid0905.020441

**Published:** 2003-05

**Authors:** Marita Noguera-Obenza, Theresa J. Ochoa, Henry F. Gomez, M. Lourdes Guerrero, Irene Herrera-Insua, Ardythe L. Morrow, Guillermo Ruiz-Palacios, Larry K. Pickering, Carlos A. Guzman, Thomas G. Cleary

**Affiliations:** * University of Texas-Houston Medical School, Houston, Texas, USA; † Instituto Nacional de la Nutricion “Salvador Zubiran,” México City, México, D. F.; ‡ Cincinnati Children’s Hospital, Cincinnati, Ohio, USA; § Center for Disease Control and Prevention, Atlanta, Georgia, USA; ¶ GBF-German National Research Centre for Biotechnology, Braunschweig, Germany

**Keywords:** Shiga toxin, EHEC, Escherichia coli, human milk, colostrum, EspA, research

## Abstract

Secretory immunoglobulin A (sIgA) is a primary factor responsible for preventing attachment of enteropathogens to gut epithelium in breastfeeding infants. We compared the frequency of sIgA to major surface antigens of enterohemorrhagic *Escherichia coli* (EHEC) in milk of 123 women from the United States and Mexico to determine whether regional differences existed in the frequency of antibodies to these surface antigens. In both groups of women, milk commonly has sIgA against various EHEC lipopolysaccharides, EspA, EspB, intimin, and less frequently against Shiga toxin. The study suggests that persons living in the U.S. are exposed to attaching/effacing enteropathogens more frequently than is generally assumed. The low frequency of antibodies to Stx1 (in 12% of Mexican and in 22% of U.S. samples) suggests that the rare appearance of hemolytic uremic syndrome in adults is not due to neutralization of toxin at the gut level. Only anti-EspA is found in most milk samples from both populations of women. EspA may represent a useful target for an immunization strategy to prevent EHEC disease in humans.

Enterohemorrhagic *Escherichia coli* (EHEC) produces multiple virulence factors; the most important are protein synthesis–inhibiting toxins: Shiga toxin 1 (Stx1) and 2 (Stx2). EHEC causes nonbloody diarrhea, hemorrhagic colitis, and hemolytic uremic syndrome (HUS). A large number of EHEC serotypes infect humans. In the United States, the predominant EHEC serotype associated with serious disease is *E. coli* O157:H7. HUS complicates approximately 5% to 8% of infections caused by *E. coli* O157:H7.

Virulence in EHEC reflects not only toxin production but also the pathogen’s ability to colonize the gut. Colonization by EHEC is related to the pathogen’s ability to form attaching and effacing lesions (intestinal mucosal changes seen in transmission electron microscopy and originally seen in intestines of animals infected with enteropathogenic *E. coli* [EPEC]) (*1*). The lesions are characterized by localized destruction of brush border microvilli and intimate adhesion of the bacterium to the host cell membrane. At the site of bacterial attachment, the host cell membrane forms a pedestal-like structure. Immunofluorescence microscopy has shown that the area of host cell in proximity to the bacterium contains polymerized actin, α-actinin, talin, and ezrin (*2*). In both EHEC and EPEC, the proteins that mediate this attachment are encoded in a chromosomal pathogenicity island called the “locus of enterocyte effacement” (LEE) (*3*). Secretion of LEE proteins is triggered by close contact with host cells. Once triggered to express LEE, the bacterium forms an export apparatus that includes a tube made of multimers of a protein (EspA); this surface organelle acts as a conduit between the bacteria and host cell (*4*–*6*). EspB, a protein thought to be involved in pore formation, is transferred to the host cell by this conduit and is found in both the host cell membrane and cytosol (*7*). EspB, with the aid of a second membrane lytic protein, EspD, forms pores in the host cell as part of the translocation mechanism (*8*). The EspA organelle is used to transfer the translocated intimin receptor (Tir), which is then inserted into the host cell membrane, where it binds to intimin, a bacterial outer membrane protein (*9*,*10*) and triggers the host cytoskeletal events that lead to attaching/effacing lesion formation.

Development of specific immunity to these antigens plays a role in protecting against infection. Immune responses are elicited in patients who are infected with EHEC or EPEC. Children infected with EPEC have been shown to have serum immunoglobulin (Ig) G against intimin, EspA, and EspB (*11*). Likewise, serum IgG against Tir, intimin, EspA, and EspB has been demonstrated during EHEC infection (*12*–*14*). Specific IgG against O157 lipopolysacchartide (LPS) (*15*) and against EHEC intimin (*16*) blocks adherence in vitro.

Human milk is protective against many enteropathogens. Because antibodies in milk reflect previous immunologic events in the mother’s gut, human milk is ideally suited for defining mucosal protective immunity. Lymphocytes are known to travel from the gut to the mammary gland. Human milk contains antibodies to EHEC intimin, EspA, EspB, and Tir (*17*–*20*), and to EPEC intimin (*19*). Incubation of colostrum or pooled human milk decreases EPEC adherence (*21*–*23*). Hence, the protective effect seen in vitro has been attributed to the presence of these antibodies. The role of virulence antigen–specific sIgA in protecting against EHEC has not been assessed directly. For EHEC and EPEC, as for most enteric pathogens, the best hope for disease control is through a vaccine strategy. Candidates for an EHEC vaccine might, in theory, include the surface-exposed components, secreted components, or both.

Antibodies in human milk can be used as an epidemiologic tool because antibodies reflect previous infection in the mother (*24*). We compared milk samples of women from Mexico and from the United States for antibodies to LPS and virulence proteins involved in the initial bacteria-host cell interaction. We also evaluated milk samples for antibodies to Stx1, a major secreted virulence factor of EHEC. We compared the frequency and amount of antibodies to each of the antigens in these two populations to determine whether important differences in sIgA antibodies exist that might provide insight into exposure to these antigens and potential protective mechanisms.

## Methods

### Population and Milk Collection

Human milk samples were collected after informed consent from 123 women living in two widely separated areas of North America: Mexico City and Norfolk, Virginia. None of the women had premature infants. The study was approved by the Institutional Review Boards of each participating institution. None of the women were known to have an underlying illness. Whether they had previously experienced infections with attaching/effacing organisms was unknown. Milk samples were obtained by using an Ameda Egnell pump (Hollister, Inc., Libertyville, IL). Samples were stored at –70°C after collection. Human milk samples were thawed and centrifuged at 13,200 rpm three times to obtain a clear fraction.

### Preparation of Antigens

#### *E. coli* LPS

*E. coli* O157:H7 LPS was extracted with phenol water by using the method described by Westphal and Jann (*25*). Other purified LPS (O26, O55, O111, O127, and O128) were obtained from Sigma (Aldrich Corp., St. Louis, MO).

#### Stx

Stx1 was purified from *Shigella dysenteriae* serotype 1 as previously described (*26*). We evaluated antibodies to Stx1 rather than to Stx2 because approximately 90% of EHEC produce Stx1. Stx2 appears to be less immunogenic than is Stx1.

### EspA and EspB

*E. coli* M15 with the plasmids encoding either C terminal histidine-tagged EspA or histidine-tagged EspB cloned from EHEC *E. coli* O26:H- strain 413/89-1 (*6*) was grown in Terrific broth (ENE Mate, ISC Bioexpress; Kaysville, UT) until optical density (OD) at A_600_ was 0.7. Bacteria were induced by adding isopropyl-β-D-thiogalactopyranoside (IPTG) to a final concentration of 1 mM at 30°C for 3 h. For purifying EspB, phenylmethylsulfonyl fluoride (1 mM) was added to the culture media. The cells were harvested by centrifugation. The resulting pellet was lysed through the addition of lysozyme and sonication. The resulting supernatant was mixed with nickel nitriloacetic agarose (Qiagen, Inc., Valencia, CA) for 1 h at 4°C. The agarose was then poured into a column and washed with increasing concentrations of imidazole in phosphate buffer (10 mM) to elute the purified proteins. All steps during the purification were performed under nondenaturing conditions following manufacturer’s instructions. Sodium dodecyl sulfate–polyacrylamide gel electrophoresis (SDS-PAGE) was performed to confirm purity of eluted proteins.

### Intimin Gamma

The 281 amino acid C terminal (extracellular) portion of intimin (C_281_γ) was cloned from *E. coli* O157:H7 strain 86-24 by using as forward primer 5-GATC- AAACCAAGGCCAGCATTACTGAGATT and reverse primer 5-AATAATTATGCCC- CGACTAAAACA. The *taq* polymerase amplified segment was inserted into polymerase chain reaction T7 NT-TOPO so that six histidine residues were added to the N terminus. The sequence was verified by digestion with *Eco*RI and *Bam*HI and automated sequencing by using dye-terminator chemistry (BigDye as the fluorescent marker) in an ABI PRISM model 377 Genetic Analyzer (Applied Biosystems, Foster City, CA. The plasmid was then inserted into BL21(DE3)pLysS and expression inducted with IPTG. After partial purification with nickel nitriloacetic agarose chromatography, the amplified protein was detected on immunoblots (as described below). The C_281_γ was located on SDS-PAGE by size, intensity of the band after IPTC induction, and confirmation that the band contained 6 x His by Western blot.

### Specific sIgA Determination by Enzyme-Linked Immunosorbent assay (ELISA)

ELISA was used to determine the presence and amount of sIgA against Stx1, EspA, EspB, and each LPS. The amount of sIgA was estimated based on the mean of duplicate measures of OD of the antigen-coated wells minus the control background wells. For each ELISA all samples were run on the same day to eliminate day-to-day variation. For EspA, EspB, and each of the LPS, 96-well polystyrene plates were coated overnight at 4°C with 1 μg/well of each antigen in carbonate buffer (pH 9.6). After coating, plates were blocked with 200 μL of 5% bovine serum albumin (BSA) in 10 mM sodium phosphate-buffered saline (PBS) for 1 h at 37°C. After each step, plates were washed five times with PBS containing 0.05% Tween 80. Human milk samples diluted 1:20 in 1% nonfat dry milk in PBS were incubated at 37°C for 1 h. Goat anti-human sIgA conjugated to horseradish peroxidase (Cappel Division of Organon Teknika, Durham, NC) was added after washing. Hydrogen peroxide with o-phenylenediamine dihydrochloride was used for color development. The reaction was stopped by adding 2 N sulfuric acid, and plates were read at 490 nm. For Stx1, we used a variation of a previously described receptor binding ELISA (*27*). Polyvinyl chloride plates were coated with Gb3, blocked with 5% BSA-PBS, washed five times with PBS-Tween, and coated with 1 μg/well of toxin overnight at 4°C. The plates were washed five times with PBS-Tween and blocked with 5% BSA-PBS before 100 μL of milk sample per well diluted 1:20 with 1% nonfat dry milk in PBS was applied. Goat anti-human sIgA conjugated to horseradish peroxidase was added after washing and OD determined as above.

### SDS-PAGE and Western Blot Analysis

SDS-PAGE was performed on the protein samples diluted in sample buffer (2-mercapthoethanol, SDS, and 0.1% bromophenol blue), boiled for 5 min, and loaded into 12.5% gels. The protein bands were visualized by staining with Coomassie blue. After SDS-PAGE, unstained protein antigens or LPS were transferred electrophoretically onto nitrocellulose membranes and blocked with 3% nonfat dry milk in Tris buffer for 1 h. The membranes were incubated overnight with the human milk samples diluted 1:20 with 1% nonfat dry milk in Tris buffer at 4°C. After three washes, the bound sIgA was detected by using peroxidase conjugates as above with chloro-1-naphthol as the color reagent. Western blot was used to determine the ELISA sensitivity and specificity as well as to detect sIgA to C_281_γ.

### Statistical Analysis

Chi-square or Fisher exact test was used to compare the frequency of sIgA-positive milk samples in the two populations for each antigen. Since anti-C_281_γ antibody was detected by immunoblot rather than ELISA, its relationship to other antibodies was determined by chi-square test or Fishers exact test. For other antibodies that were measured quantitatively, differences between the populations in amounts of antibodies were determined by a two-tailed Mann-Whitney test. The correlation between the amount of sIgA for different antigens was determined by linear regression of ELISA ODs. Because of the multiple comparisons made, differences were considered significant at p<0.01.

## Results

### Description of Populations

Milk samples from 73 women in Mexico City and 50 women in Norfolk, Virginia, were studied. No difference existed in the timing of collection of the milk samples between the two populations (Table 1).

**Table 1:** Distribution of milk samples over duration of lactation in the two study cohorts

No. days of lactation	Mexico (%)	United States (%)
<5	16 (22)	11 (22)
6–30	43 (59)	30 (60)
>30	14 (19)	9 (18)
Total	73 (100)	50 (100)

### Validation of Assays

Purification of histidine-tagged EspA and EspB resulted in a single band of protein in the eluate as visualized by SDS-PAGE. As expected, ELISAs were highly reproducible. For example, the correlation between OD_490_ on repeat assays of randomly chosen samples for antibody to O111 was 0.858 (p<0.001), and for antibody to O26, the correlation was 0.807 (p<0.001). Based on the Western blot studies, the cutoff for a positive ELISA for each antigen was considered to be an OD>0.1 at 490 nm. The sensitivity and specificity of the ELISA for various antigens were high as determined by using Western blot as the standard. For example, sensitivity and specificity were 82% and 71% for O157 LPS, 100% and 88% for EspB, and 93% and 100% for EspA, respectively. Because of the inherent differences in sensitivity of immunoblots compared to enzyme immunoassays on plastic plates, by using Western blot as the standard for defining ELISA cutoff, we may have underestimated somewhat the frequency of milk samples that contain sIgA to some antigens.

### Prevalence of Antibodies to EHEC Antigens

Large variations occurred in frequencies of milk samples containing antibody to the various LPS. The percentages of milk samples that contained antibodies to various LPS types were similar in the two populations, with the exception of anti-O128, which occurred significantly more often in milk samples from women from the United States (Table 2). However, the amount of antibody to the various LPSs differed significantly in the two settings. Mexican women had higher levels of antibodies (Table 3) against O55 and O127; they also had higher antibody levels against EspA and EspB. Milk samples from U.S. women had higher levels of antibodies against O26, O111, O128, and Shiga toxin.

**Table 2:** Comparison of prevalence of secretory immunoglobulin A to enterohemorrhagic *Escherichia coli* antigens in milk samples collected from women from Mexico and the United States

Antigen	Mexico (%) (N=73)	United States (%) (N=50)	p value
O26	58 (79)	43 (86)	NS^a^
O55	35 (48)	16 (32)	NS
O111	44 (60)	31 (62)	NS
O127	42 (58)	19 (38)	NS
O128	7 (10)	20 (40)	<0.0002
O157	25 (34)	18 (36)	NS
Stx	9 (12)	11 (22)	NS
EspA	68 (93)	45 (90)	NS
EspB	32 (44)	14 (28)	NS
Intimin (C_281γ_)	20 (27)	16 (32)	NS

^a^NS, not significant by chi-square test.

**Table 3:** Comparison of quantity of anti-enterohemorrhagic *Escherichia coli* secretory immunoglobulin A in milk samples collected from women from Mexico and the United States [median and (range) optical density_490_]^a^

Antigen	Mexico (N=73)	United States (N=50)	p value
O26	0.143 (0.016–0.305)	0.203 (0.030–0.349)	<0.002
O55	0.096 (0–0.411)	0.057 (0–0.340)	<0.006
O111	0.126 (0–0.390)	0.143 (0–0.413)	NS
O127	0.124 (0–0.416)	0.072 (0–0.350)	<0.004
O128	0.026 (0–0.228)	0.079 (0.003–0.293)	<0.0000
O157	0.061 (0–0.470)	0.050 (0–0.260)	NS
Stx	0.027 (0–0.470)	0.043 (0–0.279)	<0.007
EspA	0.283 (0.063–0.666)	0.201 (0.015–0.490)	<0.005
EspB	0.071 (0–0.546)	0.021 (0–0.430)	<0.002

^a^Mann-Whitney test for differences in amounts of antibodies for the two populations; all samples tested at 1:20 dilution; NS, not significant.

### Relationship of sIgA Antibodies to LSP, Intimin Gamma, EspA, EspB, and Shiga Toxin

Women in both populations who had antibodies to EspB nearly always had antibodies to EspA in their milk; 98% of those whose milk samples were positive for anti-EspB antibodies were also positive for anti-EspA antibodies, whereas those who were positive for anti-EspA were positive for anti-EspB 43% of the time (p<0.01 by chi square). Although anti-EspB was found less often than anti-EspA, the amount of anti-EspB correlated with the amount of anti-EspA in both populations (Tables 4 and 5). The amount of anti-EspA antibodies also was correlated with anti-O55 in milk samples from Mexico and with anti-O55 and anti-O127 milk samples from the United States. The data regarding antibodies to antigens that are EHEC-specific suggested that O55 and O111, but not O157, are important EHEC serotypes in Mexico (Table 4) since anti-Stx1 correlated well with antibodies to these LPS types. These findings are consistent with studies of meat samples in Mexico, which suggest that O157 is rarely found (*28*). Anti-C_281_ γ correlated with anti-Stx1 and anti-O55 in Mexican women but not in U.S. women (Table 6). Most milk samples (17 [85%] of 20) positive for anti-C_281_γ were from Mexican women with antibody to O55 LPS (p=0.0001). These relationships imply that Shiga toxin and intimin gamma antibodies were linked to *E. coli* O55 infection. In the milk samples from U.S. women, anti-Stx did not correlate with any LPS type including O157 (Table 5).

**Table 4:** Correlations in amount of antibodies in human milk from women in Mexico to various enterohemorrhagic *Escherichia coli* antigens (correlation/p value)

Antigen	EspA	EspB	Stx1
EspB	0.405/<0.001		
Stx1	0.242/NS^a^	0.000/NS	
O26	0.161/NS	0.124/NS	0.195/NS
O55	0.303/<0.01	0.046/NS	0.310/<0.01
O111	0.235/NS	0.069/NS	0.358/<0.001
O127	0.164/NS	0.115/NS	0.232/NS
O128	0.291/NS	0.202/NS	0.002/NS
O157	0.056/NS	0.133/NS	0.131/NS

^a^NS, not significant.

**Table 5:** Correlations in amount of antibodies in human milk from women in the United States to various enterohemorrhagic *Escherichia coli* antigens (correlation/p value)

Antigen	EspA	EspB	Stx1
EspB	0.464/<0.001		
Stx1	0.277/NS^a^	0.182/NS	
O26	0.276/NS	0.186/NS	0.165/NS
O55	0.425/<0.01	0.304/NS	0.054/NS
O111	0.380/NS	0.330/NS	0.200/NS
O127	0.470/<0.001	0.056/NS	0.262/NS
O128	0.300/NS	0.002/NS	0.135/NS
O157	0.214/NS	0.142/NS	0.068/NS

^a^NS, not significant.

**Table 6:** Relationship between presence of antibody to Intimin gamma (C_281_γ) and antibodies in human milk to various enterohemorrhagic *Escherichia coli* antigens^a^

Antigen	Mexican women	U.S. women
EspA	NS	NS
EspB	NS	NS
Stx1	<0.01	NS
O26	NS	NS
O55	<0.0001	NS
O111	NS	NS
O127	NS	NS
O128	NS	NS
O157	NS	NS

^a^p values shown for association with antibodies by chi-square analysis; NS, not significant.

## Discussion

The specific antibodies that may be important in sIgA-mediated passive immune protection and infection-induced active immunity in human milk are not known. Milk, because it contains the infection-triggered active mucosal immune response of the mother, reflects antibodies that are relevant to clearing her particular infection and to subsequently protecting her infant. As such, milk antibodies indicate to which antigens the immune system has been most responsive. Focusing on such antigens may suggest candidates for vaccine development.

In EPEC, formation of the attaching/effacing lesion is central to pathogenesis. Colostrum, and in particular, the sIgA fraction, has been shown previously to inhibit localized adherence of EPEC (*21*,*22*). Epidemiologic data also support the importance of attaching/effacing lesion formation in the pathogenesis of EHEC. The *eae*A gene is more commonly found in human isolates than bovine EHEC isolates (*29*,*30*) and in isolates known to have caused severe human diseases (*31*), suggesting that proteins found in LEE are important virulence factors.

Previous studies of anti-attaching/effacing antibodies in human milk (*17*,*19*,*22*,*32*) have reported data on small numbers of milk samples, sometimes by using only pooled colostrum or by using crude antigens. The methodologic differences between previous studies and the current data are important to interpretating the data. The most comparable previous study in the literature is that of Parissi-Crivelli et al. (*19*). They found antibodies in the colostrum of 21 Mexican women against EspB in 57%, EspA in 76%, and intimin in 81%, compared with our findings of 44%, 93%, and 27%, respectively. The differences are important because the earlier data suggest that intimin is recognized much more frequently than our results show. Routine recognition of intimin would suggest that mucosal immunity that occurs naturally during infection targets this antigen. As such, intimin might be a useful antigen for a potential vaccine development plan. However, the difference between the studies may have occurred because we determined antibodies only to the extracellular C terminal 281-amino acid portion of intimin gamma that defines tissue tropism (*33*) rather than to the whole molecule. Antibodies to the intracellular portion of intimin were not detected by using our approach. That antibodies to the intracellular portion of intimin are relevant to protection is biologically implausible. In fact, antibodies to the N terminal two-thirds of the intimin molecule do not prevent EHEC from attaching to HEp2 cells (*16*). We have therefore focused on antibodies to the receptor-binding domain (*34*) that could be relevant to protection in the gut. This approach makes our observations more pertinent to intimin gamma-positive EHEC than to organisms that express other intimin variants.

This study supports the previous suggestion that human milk can be used as an epidemiologic tool (*24*). Because lymphocytes travel from the gut to mammary glands by the common mucosal immune system, sIgA in human milk reflects previous intestinal infection. Many insights into antigen-specific sIgA, the most relevant antibody to protection from intestinal infection, can be gained by studying human milk. For example, our data show regional variations in exposure to *E. coli* LPS types in two study sites. Exposure to multiple LPS types, including O55 and O111, correlates with anti-EspA in the United States, while in Mexico only O55 occurs commonly enough for anti-EspA to correlate with anti-LPS. The lack of correlation between the presence of antibodies against Stx1 and O157 LPS in the United States suggests that mucosal immunity to the toxin is not related to previous exposure to O157 EHEC. In Mexico, the primary stimulus for development of antibody to Stx1 may be becoming infected with O55 or O111 EHEC rather than with O157 EHEC. That these serogroups are infrequently associated with HUS suggests that they may be less virulent, less easily diagnosed, or less likely to cause outbreaks of disease than *E. coli* O157:H7. The lack of readily available screening methods for EHEC serotypes other than O157 may cause the frequency of non-O157 types to be underestimated. The surprisingly low frequency of sIgA against Shiga toxin suggests that mucosal immunity to the toxin is not the basis for the low frequency of HUS in adults; antibodies with expressed virulence factors that block attachment are probably more important.

We thought that milk samples from the U.S. women would rarely show evidence of immunity to antigens expressed by EPEC or EHEC. In fact, the data suggest that exposure to organisms that produce attaching/effacing lesions must be much more common than anticipated. Antibodies to surface antigens of EHEC, particularly those involved in the initial interaction of bacteria with intestinal epithelial cells, frequently are found in human milk. The data suggest that most women have been exposed to bacteria-expressing proteins that mediate the attaching/effacing phenotype, whether these women live in Mexico City or Norfolk, Virginia. Stool survey data also suggest that these infections may be occurring more often than commonly assumed. Bokete et al. analyzed stools from 445 children in the United States and found that 5.6% shed non-O157:H7 *eae*A+ *E. coli* (*35*). A multicenter prevalence study on the cause of outpatient pediatric nondysenteric diarrhea in the United States showed that 2.7% had *E. coli* with localized adherence phenotype or with a positive probe for EPEC (*36*). Most U.S. laboratories do not routinely evaluate pediatric diarrheal stools for the presence of EPEC or EHEC. The sIgA antibody data shown here, coupled with the stool survey data, suggest that organisms producing the attaching/effacing lesion must be common pathogens in the United States. The similarity between frequencies of antibodies to important surface antigens suggests that the prevalence of HUS in industrialized countries as opposed to developing countries (*37*) is not due solely to differences in frequency of exposure to EPEC.

Our studied showed that EspA was found in most milk samples (>90%), while Parissi-Crivelli found a much lower frequency (*19*). The difference may exist because of their definition of a positive ELISA for EspA; they arbitrarily set an OD >0.2 as positive, while we established a cutoff by immunoblot. The difference could also reflect the antigens used for ELISA. We used purified EspA, which was confirmed by SDS-PAGE to be a single band with no detectable contaminants, while Parissi-Crivelli used sonicates of organisms expressing an unknown amount of EspA on a plasmid and subtracted as background the sonicates of the vector bacteria lacking the gene for EspA. We studied titers at a 1:20 dilution. Although the relatively low titers detected could reflect exposure to related antigens produced by other bacteria, the immunoblots demonstrated that the antibodies did react with the specific antigens.

Why the immune system recognizes one antigen more often than another when both are expressed during infection is not clear. However, given current understanding of the virulence mechanism involved in producing attaching/effacing lesions, the secretory IgA data are readily understandable. The lower frequency of antibodies to EspB than to anti-EspA reflects that the immune system has better recognition of the multimeric surface-exposed EspA organelle. EspB, a protein that is directly injected into the cytoplasmic membrane of intestinal epithelial cells through the EspA organelle, is likely to be less available for uptake by antigen-presenting cells.

In summary, antibodies against LEE-encoded proteins were common in samples of human milk from our two groups. Because of the structural similarity among EspA variants from multiple pathogens and the high frequency of anti-EspA antibodies, cross-reactive antibodies against EspA may provide broad cross-protection against multiple serotypes. EspA is more conserved than other virulence antigens; most clone 1 EPEC have identical EspA types, while most clone 2 are nearly 95% identical (*38*)*.* EspA in *E. coli* O127:H6 and *E. coli* O157:H7 are 85% identical (*39*) EspA is surface expressed and multimeric. Antibody to EspA is present more often than is antibody to other surface proteins. Natural exposure to EspA appears to elicit a good immune response that is long lasting as reflected by the high percentage of women who have anti-EspA in their milk samples. Unlike antibodies to LPS or intimin (*40*) that may protect against a very limited group of enteropathogens, antibodies to EspA might be able to block attachment by both EPEC and EHEC of many serogroups and thereby provide broad cross-protection. EspA may be a useful candidate for an immunization strategy that could lead to a vaccine that protects against both EHEC and EPEC of multiple serotypes.
